# Ionogels Obtained by Thiol-Ene Photopolymerization—Physicochemical Characterization and Application in Electrochemical Capacitors

**DOI:** 10.3390/molecules26030758

**Published:** 2021-02-02

**Authors:** Agnieszka Marcinkowska, Piotr Gajewski, Katarzyna Szcześniak, Mariola Sadej, Aneta Lewandowska

**Affiliations:** Institute of Chemical Technology and Engineering, Poznan University of Technology, Berdychowo 4, 60-965 Poznan, Poland; piotr.gajewski@put.poznan.pl (P.G.); katarzyna.szczesniak@put.poznan.pl (K.S.); mariola.sadej@put.poznan.pl (M.S.); aneta.b.lewandowska@doctorate.put.poznan.pl (A.L.)

**Keywords:** gel polymer electrolytes, electrochemical capacitor, ionogels, thiol-ene photopolymerization, mechanical properties

## Abstract

Flexible ionogels with good mechanical properties were obtained in situ by thiol-ene photopolymerization of trimethylolpropane tris(3-mercaptopropionate) (TMPTP) and 1,3,5-triallyl-1,3,5-triazine-2,4,6(1H,3H,5H)-trione (TATT) (with C=C: SH ratio 1:1) in four imidazolium ionic liquids (1-ethyl-3-methylimidazolium bis(trifluoromethylsulfonyl)imide—EMImNTf_2_, 1-ethyl-3-methylimidazolium trifluoromethanesulfonate-EMImOTf, 1-butyl-3-methylimidazolium bis(trifluoromethylsulfonyl)imide-BMImNTf_2_, and 1-butyl-3-methylimidazolium trifluoromethanesulfonate—BMImOTf) used in the range 50 to 70 wt.%. The mechanical and electrochemical properties of obtained ionogels were examined. Ionogels with ionic liquids (ILs) with NTf_2_^−^ anion are more puncture resistant than with OTf^−^ anion. Moreover, ionogels with the NTF_2_^−^ anion have better electrochemical properties than those with the OTf^−^ anion. Although it should be noted that ionogels with the EMIm^+^ cation have a higher conductivity than the BMIm^+^. This is connected with intermolecular interactions between polymer matrix and IL related to the polarity of IL described by the Kamlet-Taft parameters. These parameters influence the morphology of the polymer matrix (as shown by the SEM micrograph), which is formed by interconnected polymer spheres.

## 1. Introduction

The development of civilization, with its increasing demand for electricity, is contributing to the development of energy storage devices, like electrochemical capacitors (EC), also called supercapacitors. ECs are long-life energy storage systems designed for high-power applications requiring many fast charge/discharge cycles like energy recovery/capture from braking or lifting (automobiles, trains, cranes, forklifts, etc.), in peak power generation to start automobiles, burst-mode power delivery, etc. [1,2,3].

ECs consist of the electrode material, the electrolyte and the separator. Electrolytes influence properties such as capacity, thermal stability, energy and power density, life cycle [4] and they can be classified in two groups: (i) solid/quasi-solid-state (SPE) which include dry solid polymer electrolytes [5], gel polymer electrolytes (GPE) [6,7,8,9], polyelectrolytes [10,11] and (ii) liquid electrolytes which include organic, ILs- and water-based electrolytes [4]. IL electrolytes can operate in a wide potential window of approximately 3.0–4.0 V [4] and have high thermal and chemical stability, low vapor pressure, moreover they are non-inflammable because such electrolytes do not contain easily flammable solvents, such as acetonitrile. This makes ILs safe electrolytes even for high-temperature applications [12].

Another important part of the EC is the separator, which must separate the positive and negative electrodes to prevent electrical short circuits. At the same time, it should enable quick transport of ions between the electrodes. The separator may be a porous membrane (for liquid electrolytes) or a self-standing solid electrolyte (that plays a dual role of electrolyte and separator) [13]. It should allow fast transport of ions between the electrodes [13], have low thickness and high porosity and display high conductivity. Moreover, it should have good mechanical properties as well as should be quickly and easily wetted by the electrolyte. In the case of flexible energy storage devices, electrolyte leakage from the device should be avoided [3].

Gel-polymer electrolytes with ILs as a conductive component, i.e., ionogels, meet the requirements of safety and flexibility [14]. They can be prepared by two main methods: doping and polymerization process. Doping methods include solution-cast methods (mixing solvents) and impregnation methods (swelling the polymer with the IL). These methods do not allow control of the composition of the polymer used (only commercially available polymers can be used). Additionally, the dissolution of the polymer and subsequent evaporation of the solvent is highly time and energy consuming what places high limitations on the commercial application of this process [4]. Direct polymerization of monomers dissolved in ILs seems the most advantageous approach, thus avoiding the use of additional solvents. In addition, using monomers with various structures allows for the precise design of the matrix’s final properties. In free radical polymerization, no by-product is obtained but depending on the initiation method, the process can take from a few hours (thermal and redox initiation) to a few seconds/minutes (photoinitiation) [15]. Moreover, the use of UV radiation to initiate the polymerization process allows one to perform it at ambient temperature with low energy consumption [15]. Therefore, photopolymerization seems to be a good choice for obtaining polymeric ionogels. The thiol-ene matrix is interesting choice, as we reported in our previous work [16], which is connected with additional advantages such as reduced or no oxygen inhibition and formation of a polymer with homogeneous network [17]. Thiol-ene polymerization consists in stoichiometric addition of a thiol group (SH) to a carbon-carbon double bond (C=C) [18]. The addition of thiols to enes shares many of the attributes of click reactions. The concept of click chemistry is gaining popularity, due to its highly reliable and selective reactions. In click chemistry, the reaction must be modular, wide-ranging and give very high yields with a variety of starting materials; the byproduct must be easily removed (like crystallization or distillation, without requiring chromatographic methods) and must be able to be applied in both small and large-scale production settings [17,18,19,20,21,22]. Research and development in this field are increasing exponentially, its applications are increasingly found in all aspects of drug discovery and delivery, ranging from metal catalyzed azide/alkyne click reaction through combinatorial chemistry and target-templated in situ chemistry, to proteomics and DNA research, using bioconjugation reactions [23,24,25,26,27]. Click reactions are also used in polymer science and material engineering, for both polymer/materials synthesis and for modification [22,28,29,30]. A large amount of literature, including excellent review articles, is available on this subject [17,18,31]. The highly uniform structure is ideal for rapid fabrication new polymer nanocomposite and hybrid [32,33], protective coatings and films [34], covalent functionalization of nanoparticles [28,35,36], graphene and graphene oxide [30,32], etc. Highly efficient and bioorthogonal click methodologies may provide efficient functionalization [29]. Among various click reactions (azide–alkyne Huisgen 1,3-dipolar cycloaddition, Diels–Alder addition, and thiol–ene reaction), thiol–ene click reaction is favorable for polymer synthesis: the reaction can be easily triggered by free radicals without using toxic catalysts; if the radicals are generated by photoinitiation, many parameters (e.g., light intensity, exposure dose, and duration of the photoreactions), can be easily controlled [37].

Our preliminary studies [16,38] explained the influence of ILs on the polymerization kinetics as well as on the conductivity of thiol-ene ionogels. However, the possibility of using them as gel polymer electrolytes requires appropriate mechanical properties and electrochemical characteristics. Thus, the aim of this work is to investigate the mechanical and electrochemical properties of ionogels prepared by thiol-ene photopolymerization.

## 2. Results and Discussion

Selected thiol-ene monomers for matrix synthesis of ionogels, i.e., thiol trimethylolpropane tris(3-mercaptopropionate) (TMPTP), and ene 1,3,5-triallyl-1,3,5-triazine-2,4,6(1H,3H,5H)-trione (TATT), are very reactive and polymerize with network formation [39,40]. The obtained polymer has good mechanical properties what is associated not only with monomers structure but also with a homogenous network. Photopolymerization of a TATT+TMPTP mixture in ILs allowed us to obtained ionogels by an in situ method. In our previous investigations we focused on kinetic investigations of thiol-ene reaction in ILs [16]. Now we decided to perform a wider characterization of the obtained materials in terms of their use as solid polymer electrolytes. We have developed a method of synthesizing much thinner ionogels, with a thickness off 250 μm, and examined their mechanical and electrochemical properties as a function of the IL content and its type.

**Figure 1:**
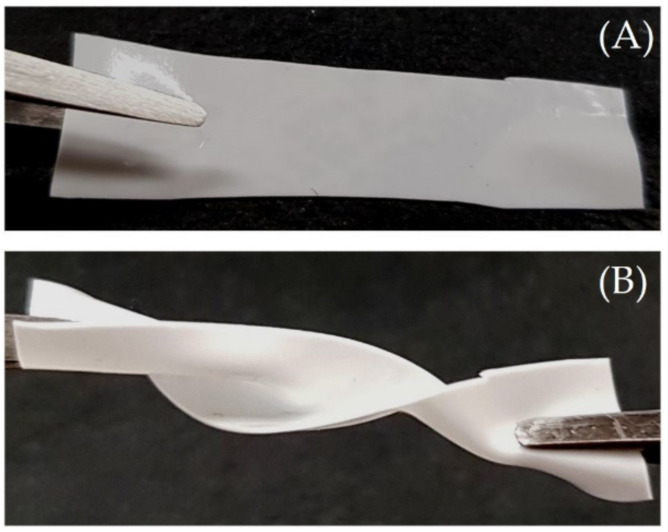
(**A**) Obtained and (**B**) twisted ionogel poly(TATT+TMPTP) with 70 wt.% of BMImOTf.

**Figure 2:**
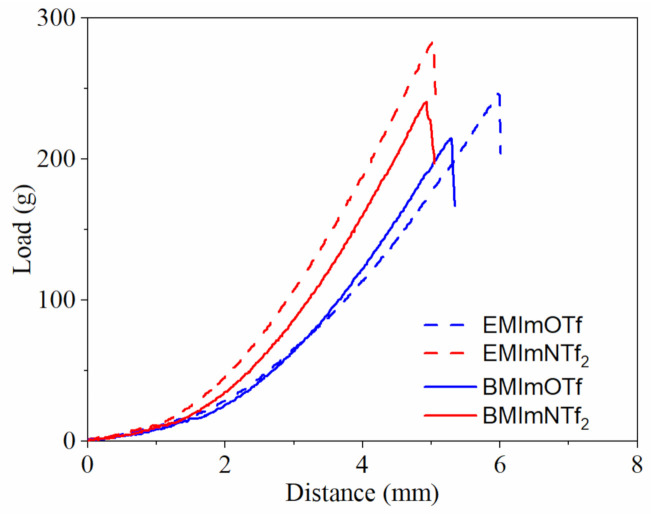
Load Load-distance curves for obtained ionogels, poly(TATT+TMPTP) with 50 wt.% of IL.

**Figure 3:**
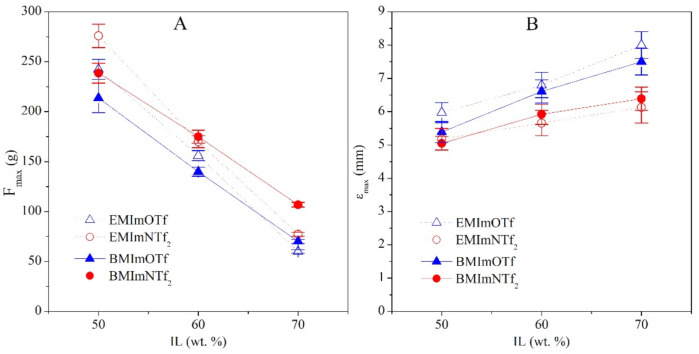
Puncture strength of ionogels: dependence of (**A**) maximum load needed to puncture the sample, *F_max_*, and (**B**) maximum elongation, *ε_max_*, on the IL content in the ionogels.

The synthesized materials are flexible and quite mechanically strong for gels, i.e., the material can be twisted or rolled up without damaging it (Figure 1). The mechanical properties of the obtained ionogels were investigated for puncture resistance. In Figure 2, exemplary load-distance curves of the obtained ionogels, poly(TATT+TMTMP) containing 50 wt.% of IL in the structure, are shown. As can be seen, the maximum load, as well as elongation, depends on the ionic liquid used for the synthesis of the ionogels. The maximum load needed to puncture the sample, *F_max_*, and maximum elongation, *ε_max_*, on the IL content in the ionogels were plotted in Figure 3. The puncture strength (*F_max_*) of synthesized ionogels with 50 wt.% of IL is in the range of 214–275 ± 14 g (for comparison, the puncture strength of commercially available cellulose paper separator NKK F3040 (Nippon Kodoshi, Kochi, Japan) with a porosity of 55% and the thickness of 40 µm is equal to 106 ± 13 g (before electrolyte soaking) and paper saturated with IL EMImNTf_2_, is equal to 42 ± 3 g). The higher concentration of IL causes deterioration of puncture strength of investigated ionogels, as expected, which is in the range of 60–107 ± 2.5 g. A more detailed insight into the obtained results shows that *F_max_* is slightly better for ionogels containing ILs with the NTf_2_^−^ anion (EMImNTf_2_ and BMImNTf_2_). Mechanical properties of investigated ionogels can be affected by several factors, i.e., intermolecular interactions between its components, polymer matrix structure as well as ionogel morphology.

The polymer matrix glass transition temperature, *T_g_*, is close to room temperature (21.4 °C), but for ionogels with 50 wt.% of IL it is below this temperature, in the range of 15–16 °C (Table 1). This indicates that the ILs swell the polymer matrix in a similar manner, causing a similar plasticizing effect regardless of the IL structure.

**Figure 4:**
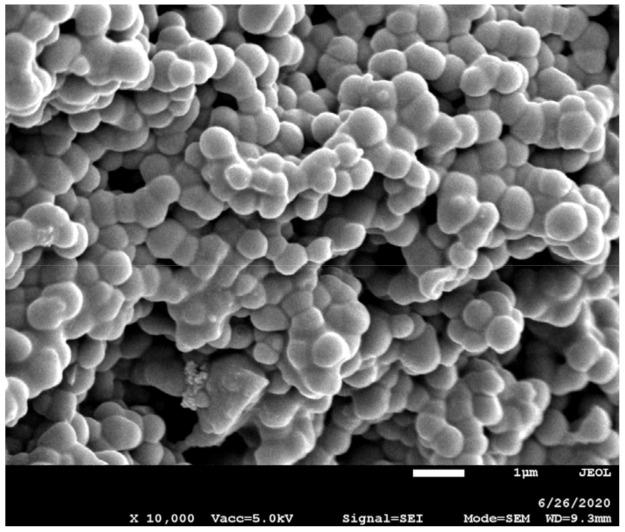
SEM micrograph of ionogel matrix obtained in presence of 50 wt.% of EMImNTf_2_.

Therefore, it seems that the differences in mechanical properties are not likely to be due to the mobility of the polymer network in different ILs. Thiol-ene photopolymerization of the study composition leads to a crosslinked polymer network swollen with IL. The polymerization reaction takes place, as can be seen from SEM micrographs (Figure 4), as dispersion polymerization, providing a connected polymer sphere morphology. The size of the obtained spheres is related to the type of IL (the chemical structure of the anion and cation) and its concentration (Table 2). Ionogels with EMImNTf_2_ and BMImNTf_2_ have more fused polymer spheres than ionogels with ILs with OTf^−^ anion. Such a polymer spheres connection can cause greater stiffness of the material and can lead to the greater puncture strength, *F_max_* (Figure 3A).

**Figure 5:**
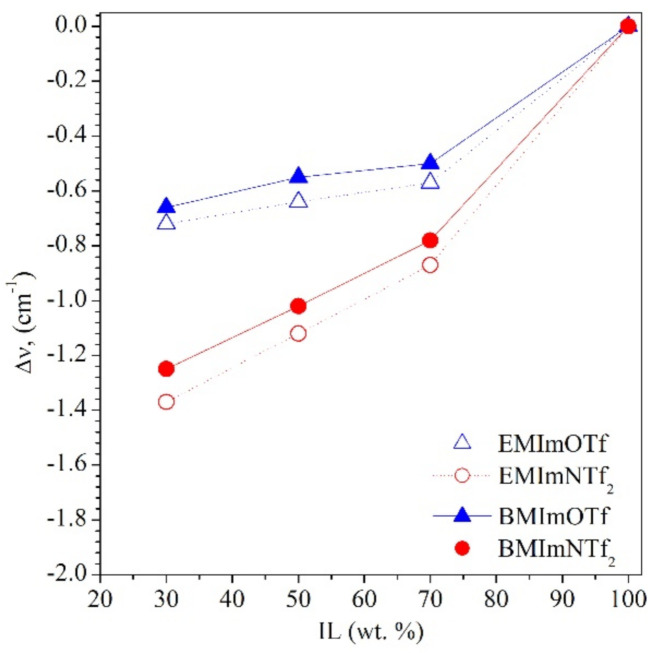
Shifts of the IR absorption band of C^2^-H bond (imidazolium ring of ionic liquid) as a function of IL concentration in ionogels. Lines are guides for eyes.

The extent of polymer coagulation during dispersion polymerization may be related, among other things, to the interaction of the IL with the polymer being formed. The strength of interactions occurring between components of ionogels, i.e., IL and polymer matrix, can be related to Kamlet-Taft parameters *(α, β* and π*) which describe the polarity of the IL, as we claimed in our previous work [16]. The *α* and *β* parameters are a measure of hydrogen bond acidity (donating ability) and hydrogen bond basicity (accepting ability) of the solvent, respectively. The π* parameter describes dipolarity/polarizability of the solvent. The H-bond basicity (*β*) depends strongly on the IL anion and H-bond acidity (*α*) is mainly determined by the IL cation, although the anion also plays a role due to the strong cation-anion interactions which reduce the ability of cation to interact with other compounds [41]. The values of *α* parameters are basically the same for all used ILs (*α*_BMImNTf2_ = 0.62, *α*_EMImNTf2_ = 0.66, *α*_BMImOTf_ = 0.63, *α*_EMImOTf_ = 0.62) [42] but *β* parameters (depending on the type of anion) for ILs with NTf_2_^−^ anion (*β*_BMImNTf2_ = 0.25, *β*_EMImNTf2_ = 0.28) [42] are lower than for ILs with OTf^−^ anion (*β*_BMImOTf_ = 0.46, *β*_EMImOTf_ = 0.48) [42]. IL interacts with polymer matrix mainly by imidazolium cation. Since the interactions of anion NTf_2_⁻ with imidazolium cation are weaker than that of anion OTf^−^ (NTf_2_^−^ has a lower value of *α*), EMImNTf_2_ and BMImNTf_2_ can interact stronger with the polymer (a greater shift of the imidazolium ring C^2^-H absorption band on IR spectra, Figure 5) what leads to a stronger coagulation (aggregation) of the polymer during dispersion polymerization. The solvency of the medium is higher.

The way the spheres connect in polymer matrix also affects the flexibility of the ionogels. The smaller the connection area of the polymer spheres is the lower the stiffness of the ionogels is, which can lead to a more flexible material. Thus the maximum elongation of the sample during a puncture test is higher for ionogels with EMImOTf and BMImOTf (Figure 3B). The highest value of the maximum elongation *ε_max_* is obtained for ionogel with EMImOTf. It may be due to the fact that the polymer formed during the polymerization in EMImOTf has the largest spheres, additionally coagulated to a small extent. This causes that spheres are connected to each other with a small contact area, which can lead to easier deformation of ionogel.

**Figure 6:**
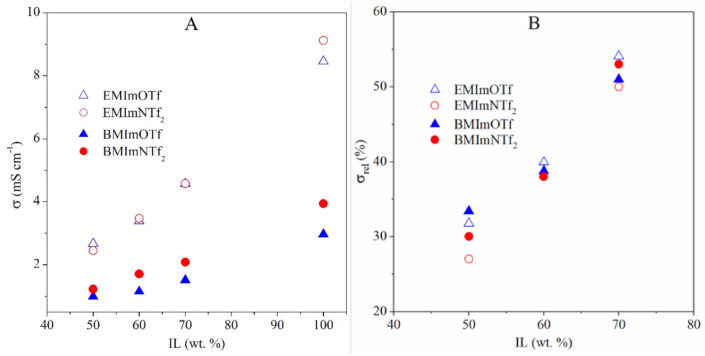
Dependence of (**A**) conductivity, *σ*, and (**B**) relative conductivity, *σ_rel_*, on the IL content in the ionogels. Residual standard deviations of *σ* and *σ_rel_* values are below 3%.

The ionogels conductivity (Figure 6A), mainly depends on the conductivity of IL used during synthesis. Ionogels with ILs containing BMIm^+^ cation show a lower conductivity value compared to ILs with EMIm^+^ cation. This observation is correlated with the lower conductivity of pure ILs containing BMIm^+^ cation. Ionogels containing ILs with the same cation BMIm^+^ but different anions (NTf_2_^−^ or OTf^−^) show slightly higher conductivity values for BMImNTf_2_-based ionogels, but in the case of EMImNTf_2_^−^ and EMImOTf-based ionogels, the difference is negligible. To better understand the influence of the ionogel composition on conductivity, the relative conductivity as a function of IL content was plotted (Figure 6B). As can be seen, for the lowest ILs content in ionogels, a slightly lower relative conductivity value is observed for ionogels with ILs containing NTf_2_^−^ anion. This behavior may be related to interactions of IL with the polymer matrix and their effects on the structure of the polymer matrix.

**Figure 7:**
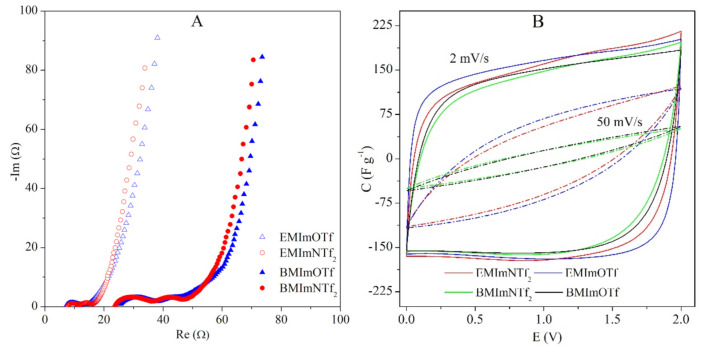
(**A**) Nyquist plot at open-circuit voltage and (**B**) CVs (at 2 and 50 mV s^−1^) of the AC/AC capacitors with the various ionogels with 70 wt.% of IL.

The Nyquist plot obtained from electrochemical impedance spectroscopy measurements is shown in Figure 7A. Furthermore, ECs containing ionogels with EMImNTf_2_ and EMImOTf are characterized by three times lower resistance (ESR and EDR) in comparison with similar ECs containing ionogels with BMImNTf_2_ and BMImOTf. At the same time, the resistance of ECs with ionogels containing ILs with the same cation but different anions is slightly lower for ILs with NTf_2_^−^ anion. The observed differences in resistance are in good agreement with conductivity of individual ionic liquids and ionogels (Figure 6A). Taking into account these data, comparable charge propagation properties may be expected for the ECs with EMImNTf_2_ and EMImOTf as well as for ECs with BMImNTf_2_ and BMImOTf.

The latter remark is confirmed by Figure 7B and Figure S1 presented in the ESI which shows almost identical CVs for the ECs containing EMImNTf_2_/EMImOTf and BMImNTf_2_/BMImOTf, respectively.

**Figure 8:**
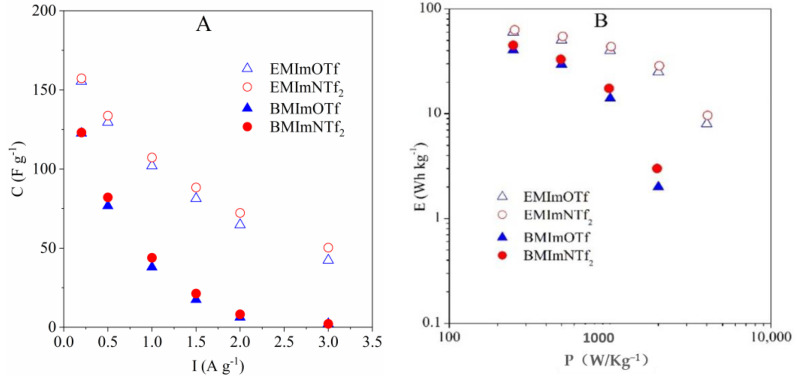
(**A**) Discharge capacitance vs. current and (**B**) Ragone plot of the ECs with various ionogels. Capacitance, energy and power calculated per mass of AC in one electrode.

Figure 8 presents the discharge capacitance and Ragone plot of the four ECs with the different ionogels. Electrochemical capacitors with ionogels containing EMImNTf_2_ and EMImOTf, compared to ECs containing ionogels with BMImNTf_2_ and BMImOTf, show higher capacitance (Figure 8A) and energy (Figure 8B) as a function of current and power, respectively. The capacitance value (Figure 8A) of ECs with EMImNTf_2_ and EMImOTf based ionogels, change from ca. 155 F g^−1^ to 50 F g^−1^ when current increase from 0.2 A g^−1^ to 3 A g^−1^, respectively. For the same current range, capacitance obtained for ECs with ionogels containing BMImNTf_2_ and BMImOTf decrease from ca. 120 F g^−1^ to 2 F g^−1^. Similar dependencies can be observed in Ragone plot (Figure 8B). At power of ca. 250 Wh kg^−1^, the energy value is equal ca. 60 Wh kg^−1^ for ECs with EMImNTf_2_ and EMImOTf and ca. 45 Wh kg^−1^ for ECs with BMImNTf_2_ and BMImOTf-based ionogels. A power increase up to 2000 Wh kg^−1^ causes an energy decrease up to 27 and 3 Wh kg^−1^ for ECs with EMImNTf_2_/EMImOTf and BMImNTf_2_/BMImOTf, respectively. Similar dependencies can be observed in the plot of capacity vs. scanning rate shown in the ESI (Figure S2). Obtained results are consistent with the conductivity of individual ionogels and the resistance data obtained on the Nyquist plots for each electrochemical capacitor.

All of these observations suggest that the ionogels can be successfully applied in the ECs. By adequate selection of IL used for ionogel synthesis, it is possible to construct ECs characterized by high capacitance and energy value, keeping all advantages of gel polymer electrolyte (no electrolyte leakage, high mechanical properties, high flexibility) at the same time. Better results of electrochemical parameters were obtained for ECs with ionogels containing ILs with EMIm^+^ cation, what is related to higher conductivity of these ILs and ionogles with these ILs.

## 3. Materials and Methods

### 3.1. Used Reagents

Monomers: trimethylolpropane tris(3-mercaptopropionate) (TMPTP), purity ≥95%, 1,3,5-triallyl-1,3,5-triazine-2,4,6(1H,3H,5H)-trione (TATT), purity 98%. Monomers were provided by Sigma-Aldrich (St. Louis, MO, USA)). Imidazolium ionic liquids: 1-ethyl-3-methylimidazolium bis(trifluoromethylsulfonyl)imide (EMImNTf_2_), 1-ethyl-3-methylimidazolium trifluoromethanesulfonate (EMImOTf), 1-butyl-3-methylimidazolium bis(trifluoromethylsulfonyl)imide (BMImNTf_2_), 1-butyl-3-methylimidazolium trifluoromethanesulfonate (BMImOTf), purity ≥99.0% (water content ≤100 ppm; halide content ≤100 ppm), were provided by Merck (Darmstadt, Germany). The photoinitiator, 2,2-dimethoxy-2-phenylacetophenone (DMPA) was purchased from Sigma-Aldrich (St. Louis, MO, USA).

### 3.2. Ionogels Synthesis

**Figure 9:**
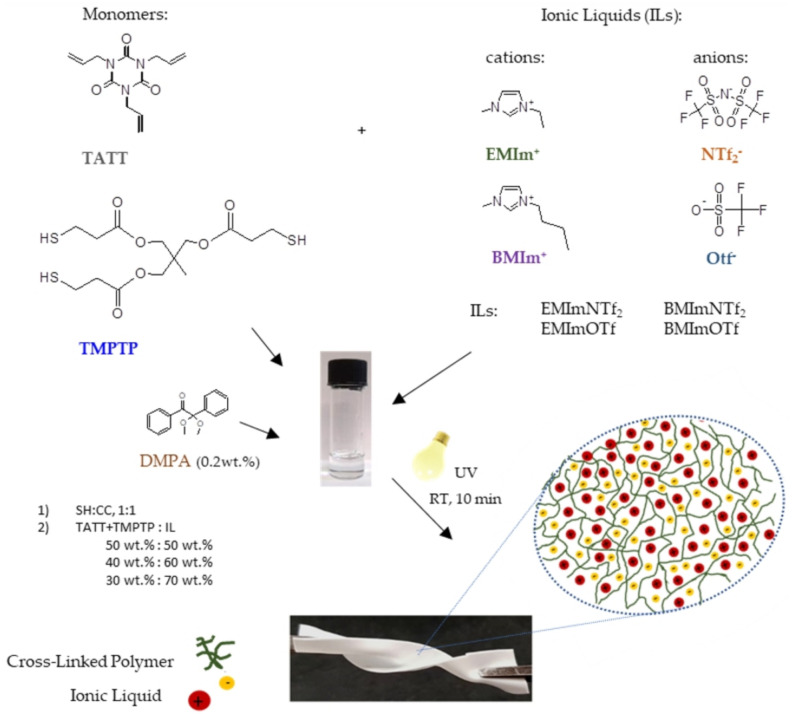
Schematic illustration of ionogels synthesis.

The concentration of ionic liquids in the photocurable compositions was 50, 60 and 70 wt.%, calculated on the whole composition. The monomers TATT and TMPTP were used in stoichiometric ratios of thiol functional groups to ene functional groups (1 : 1, SH : C=C). The concentration of photoinitiator was 0.2 wt.%, calculated based on the total composition. The samples were prepared in a glove box under a pure argon atmosphere. The composition consisted of a mixture of monomers, ionic liquid and photoinitiator was homogenized in a digital shaker and poured into 0.3 mm thick glass molds. UV irradiation was performed for 5 min on each side of the mold with ASN-36W UV lamp (*λ_max_* = 365 nm, light intensity 6 mWcm^−2^). The ionogels preparation by one-pot reactions of TATT and TMPTP in existence with ionic liquid (EMImNTf_2_, EMImOTf, BMImNTf_2_ or BMImOTf) is shown in Figure 9. Then, test samples with suitable dimensions were cut off from the obtained sheets of ionogels.

### 3.3. Physicochemical Characterization

#### 3.3.1. Scanning Electron Microscope—SEM

The images were obtained on a 7001F scanning electron microscope (JEOL, Tokyo, Japan, SEI detector, 15 kV accelerating voltage). Before measurement, the synthesized ionogels were treated with methanol and dried in an oven at 35 °C to remove the ionic liquid. Then they were placed on a stub of metal with adhesive and coated with an ultrathin gold/palladium coating, deposited on the sample by low-vacuum sputter coating.

#### 3.3.2. Puncture Resistance

In order to characterize the mechanical properties of the obtained ionogels, a puncture resistance test was conducted. The measurements were performed with a CT3 Texture Analyzer (Ametek Brookfield, Middleboro, MA, USA). A sample with a diameter of 16 mm was cut out from ionogel just after synthesis. Its thickness was measured and then the sample was fixed in a 10 mm hole diameter sample holder and tested for puncture strength using a spherical probe with 2.5 mm radius. During the measurement, the load and displacement of the measuring probe were recorded until the sample was punctured (probe displacement rate −0.3 mm∙s^−1^). Because synthesized ionogels slightly differed in their thickness, the load was normalized to a uniform thickness (250 µm) in order to compensate for the influence of sample thickness on the measurement. For each ionogel, five measurements of mechanical properties were made. Based on the obtained results, the means and standard deviations were calculated.

#### 3.3.3. Ionic Conductivity

The ionic conductivity of the ionogels was investigated by electrochemical impedance spectroscopy in the frequency range from 1 kHz to 1 MHz using the SP-300 potentiostat/galvanostat (Biologic, Seyssinet-Pariset, France). The experiment was performed in a two-electrode Swagelok^®^ type electrochemical vessel at room temperature. Current collectors were made of 316L stainless steel.

The ionic conductivity of the ionogels (*σ*) was calculated from Equation (1):

σ = l/A∙σ_S_ (1)

where *σ* is the ionic conductivity of the ionogel—S cm^−1^, *l* is the thickness of the ionogel—cm, *A* is a ionogel surface area—cm^2^ and *σ_S_* represents the volumetric conductance of the ionogel sample—S.

In addition, the relative conductivity *σ_rel_* of investigated ionogels in relation to the conductivity of an IL, were calculated from Equation (2):

σ_rel_ = σ/σ_IL_ 100% (2)

where *σ_IL_* is the ionic conductivity of IL—S cm^−1^.

#### 3.3.4. Infrared Spectroscopy (FTIR-ATR)

Infrared (IR) spectra were performed on a Nexus Nicolet 5700 Fourier Transform Infrared Spectrophotometer (FTIR, Thermo Electron Scientific Instruments Corporation, Madison, WI, USA) equipped with an attenuated total reflection (ATR) accessory with a ZnSe crystal (T = 25 °C, range 4000–600 cm^−1^, resolution 4 cm^−1^ at 64 scans). Results are presented as IR shift (*Δν*) of absorption band of the C^2^-H bond of IL imidazolium ring, i.e., as the difference between the position of the absorption peak for ionogel *(ν_ionogel_*) and pure IL (*ν_IL_*).

**Figure 10:**
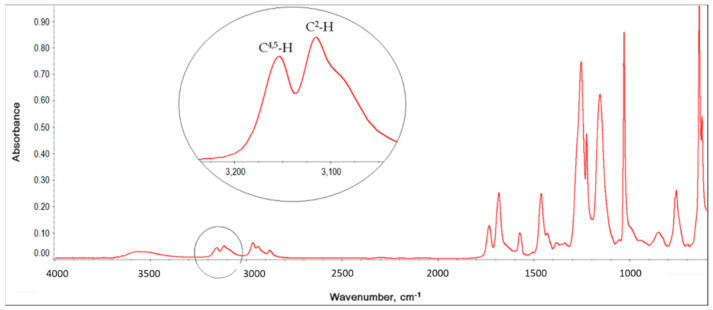
IR spectrum of ionogel poly(TATT+TMPTP) with 70 wt.% of BMImOTf.

The example of IR spectrum of ionogel poly(TATT+TMPTP) obtained in 70 wt.% of BMImOTf is shown in Figure 10. The position of the absorption band of the C^2^-H bond of the ionic liquid BMImOTf in ionogel is indicated on the spectrum.

#### 3.3.5. Differential Scanning Calorimetry—DSC

The glass transition temperature *T_g_* was measured by differential scanning calorimetry (DSC) method using a DSC1 instrument (Mettler-Toledo, Greifensee, Switzerland). Measurements were performed under a nitrogen atmosphere with a heating rate of 20 °C min^−1^ in the temperature range from −80 °C to 180 °C. *T_g_* values were evaluated from the second run of the DSC measurement.

### 3.4. Electrochemical Measurements of Capacitors

#### 3.4.1. Preparation of Electrodes

In order to compare the various ionogels in ECs, the same electrode composition was used in all experiments. The carbon electrodes were prepared by mixing an appropriate amount of activated carbon—90 wt.% of Maxsorb MSP-20X (Kansai Coke and Chemicals Co., LTD., Hyogo, Japan) with carbon black—5 wt.% of C65 (Imerys, Bironico Switzerland) and binder—5 wt.% of PTFE (60 wt.% water suspension, Sigma-Aldrich, St. Louis, MO, USA). The electrode components were mixed in deionized water until a homogenous suspension was obtained. Then, the solvent was partially evaporated and a thin film was prepared by rolling out and calendaring to an average thickness of 200 ±15 μm. Thereafter, the electrode film was glued on a stainless steel current collector using Acheson electrodag PF-407C (Henkel, Dusseldorf, Germany).

#### 3.4.2. Electrochemical Investigations

The impact of ionogels on EC properties was evaluated in a two-electrode symmetric AC/AC Swagelok^®^ cell (Swagelok Switzerland, Wohlen, Switzerland) assembly with ILs electrolyte, using 12 mm diameter electrodes and 13 mm diameter, 250 µm thick ionogels containing 70 wt.% of ionic liquid. In order to obtain precise results, all used electrodes had similar mass: 12–13 mg. Before assembling the Swagelok^®^ device the carbon electrodes were soaked with the ionic liquid, and the EC was assembled in an argon atmosphere. The cells were investigated by cyclic voltammetry (CV) with various scan rates from 1 to 50 mV s^−1^ and up to maximal cell potentials from 1 to 3 V, galvanostatic (0.1 to 3 A g^−1^) charge/discharge with potential limitation (GCPL), charge/discharge at constant power with voltage limitation between 1.5 to 3 V (CP, power in range 250 to 4000 Wh kg^−1^) and electrochemical impedance spectroscopy at OCV (EIS, over the frequency range from 1 MHz to 1 mHz with a 10 mV amplitude), using a SP-300 potentiostat/galvanostat (Biologic, Seyssinet-Pariset, France).

## 4. Conclusions

Thiol-ene photopolymerization of TMPTP and TATT in an IL was used to obtain thin, flexible, quite mechanically strong ionogels, i.e., which can be twisted or rolled-up without suffering damage. These materials are also characterized by high capacitance and energy. Thus, this method is convenient for the in-situ synthesis of ionogels which can be used as GPE. With increasing IL content in the composition, the ionic conductivity of ionogels increases, but the puncture strength decreases. Ionogels containing ILs with the NTf^−^ anion are characterized by better mechanical and electrical properties. This is related to the intermolecular interactions between the ionogel components, i.e., the IL and the polymer. These interactions affect the morphology of ionogels, the matrix of which consists of interconnected polymer spheres. The EMImNTf_2_ and BMImNTf_2_ liquids interact more strongly with the polymer matrix, which results in greater agglomeration of the polymer particles. This causes a larger connection area of polymer spheres and greater puncture resistance of ionogels, while reducing their flexibility.

## Figures and Tables

**Table 1:** Glass temperature of investigated ionogels with 50% of IL.

Poly(TATT + TMPTP)	Poly(TATT + TMPTP) + 50 wt.% of IL			
	EMImNTf_2_	BMImNTf_2_	EMImOTf	BMImOTf *
*T_g_* °C				
21.4	16.1	15.3	15.8	-

* impossible to determine due to overlapping melting point on *T_g_*_._

**Table 2:** Size of polymer spheres in synthesized ionogels.

Matrix Poly(TATT + TMPTP) with IL	*C_IL_*, wt.%	Spheres Size, µm
EMImNTf_2_	50	0.51–0.74
	70	0.24–0.40
BMImNTf_2_	50	0.37–0.56
	70	0.20–0.32
EMImOTf	50	0.50–1.00
	70	0.60–0.90
BMImOTf	50	0.30–0.53
	70	0.28–0.46

## Data Availability

Data available in a publicly accessible repository.

## Supplementary Materials

The following are available online, Figure S1: Cyclic voltammograms of AC/AC capacitors with ionogels, Figure S2: Dependence of capacitance on scan rate of AC/AC capacitors with different ionogels.
