# Supplementary material for: Ionogels Obtained by Thiol-Ene Photopolymerization—Physicochemical Characterization and Application in Electrochemical Capacitors
Source: Molecules. 2021 Feb 2;26(3):758. doi: 10.3390/molecules26030758 (PMC7867143; doi:10.3390/molecules26030758)
Supplement: Supplementary file 1 [file molecules-26-00758-s001.pdf]

Electronic supplementary information

Ionogels obtained by thiol-ene photopolymerization – physicochemical characterization and application in electrochemical capacitors

Agnieszka Marcinkowska*, Piotr Gajewski, Katarzyna Szcześniak, Mariola Sądej, Aneta Lewandowska

Institute of Chemical Technology and Engineering, Poznan University of Technology, Berdychowo 4, Poznan, 60-965, Poland, Agnieszka.Marcinkowska@put.poznan.pl

1. Cyclic voltammograms on the AC/AC capacitors

The cyclic voltammograms (CVs) of AC/AC capacitors with different iongels were recorded at increasing values of maximum cell potential (fig. S1). As it can be seen, the CVs are almost identical for ECs containing $EMImNTf_2$/EMImOTf and $BMImNTf_2$/BMImOTf respectively.

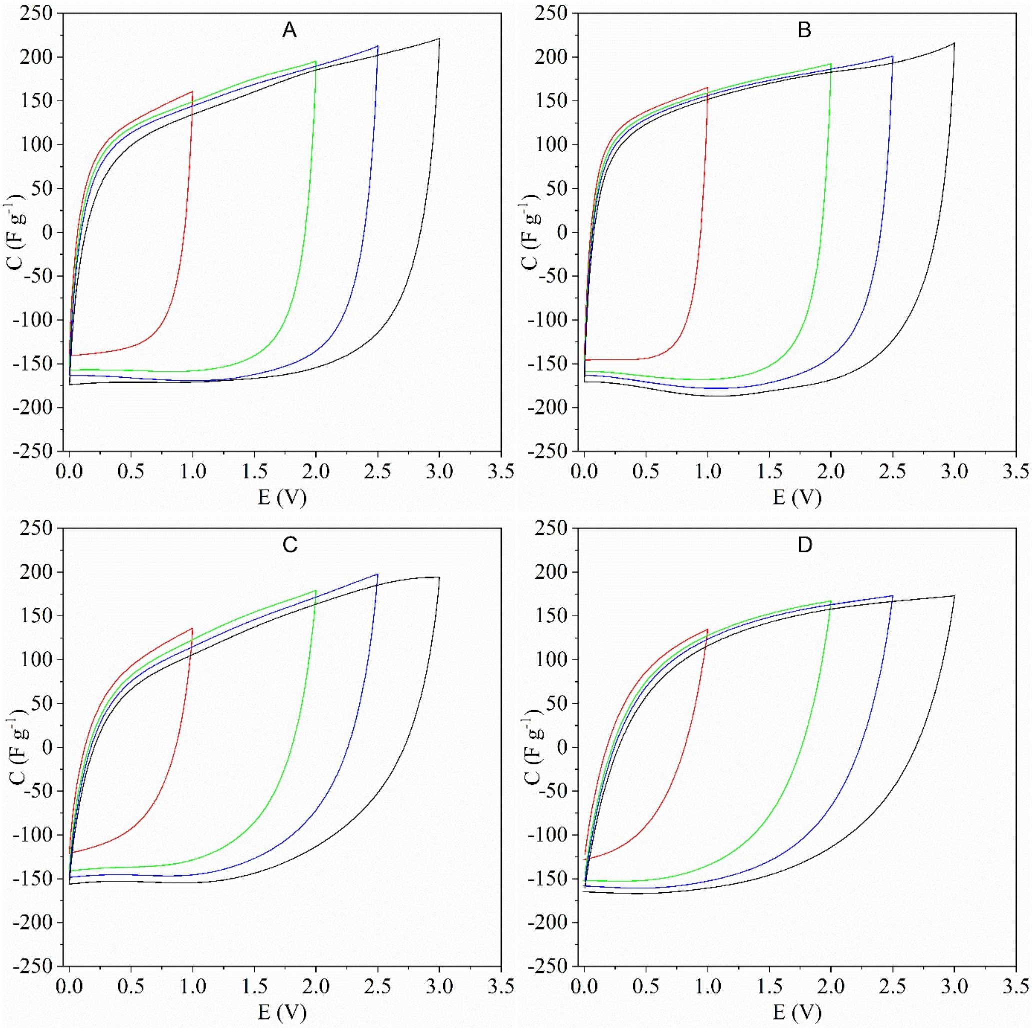

Figure S1. Cyclic voltammograms of AC/AC capacitors with iongles: (A) $EMImNTf_2$, (B) EMImOTf, (C) $BMImNTf_2$, (D) BMImOTfup to various values of maximum potential. Scan rate 5mV s^{-1}.

2. Dependence of capacitance on scan rate for AC/AC capacitors with different iongels

The cells were investigated by cyclic voltammetry (CV) with various scan rates from 1 to 50 mV s^{-1} up to maximal potential equal 2V. Based on the obtained results, the dependence of capacitance on the scan rate was plotted (fig. S2). As can be seen, decreasing of capacitances in function of scan rate is almost identical for ECs containing $EMImNTf_2$/EMImOTf and $BMImNTf_2$/BMImOTf respectively.

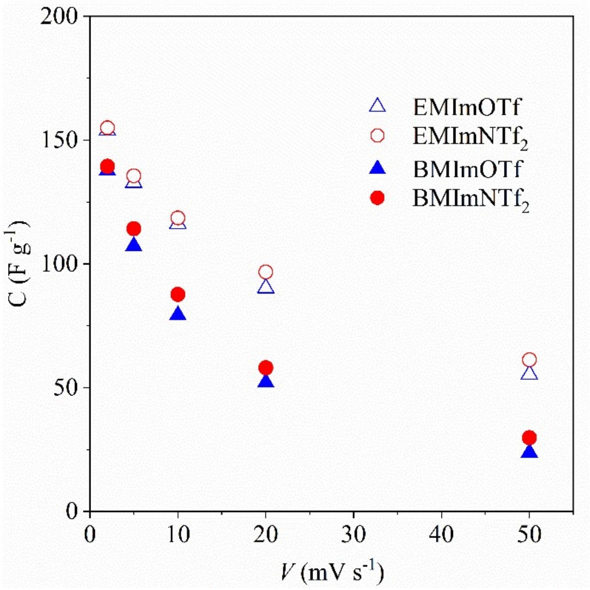

Figure S2. Dependence of capacitance on scan rate of AC/AC capacitors with different iongels.Scan rates from 1 to 50 mV s^{-1}, maximum potential 2 V.
